# Supplementary material for: Are Putative Beta-Lactamases Posing a Potential Future Threat?
Source: Antibiotics (Basel). 2025 Nov 20;14(11):1174. doi: 10.3390/antibiotics14111174 (PMC12649608; doi:10.3390/antibiotics14111174)
Supplement: Supplementary file 1 [file antibiotics-14-01174-s001.zip › Supplementary Figure S1.pdf]

Supplementary Figure S1. Genomic organization of candidate beta-lactamase loci in two bacterial species.

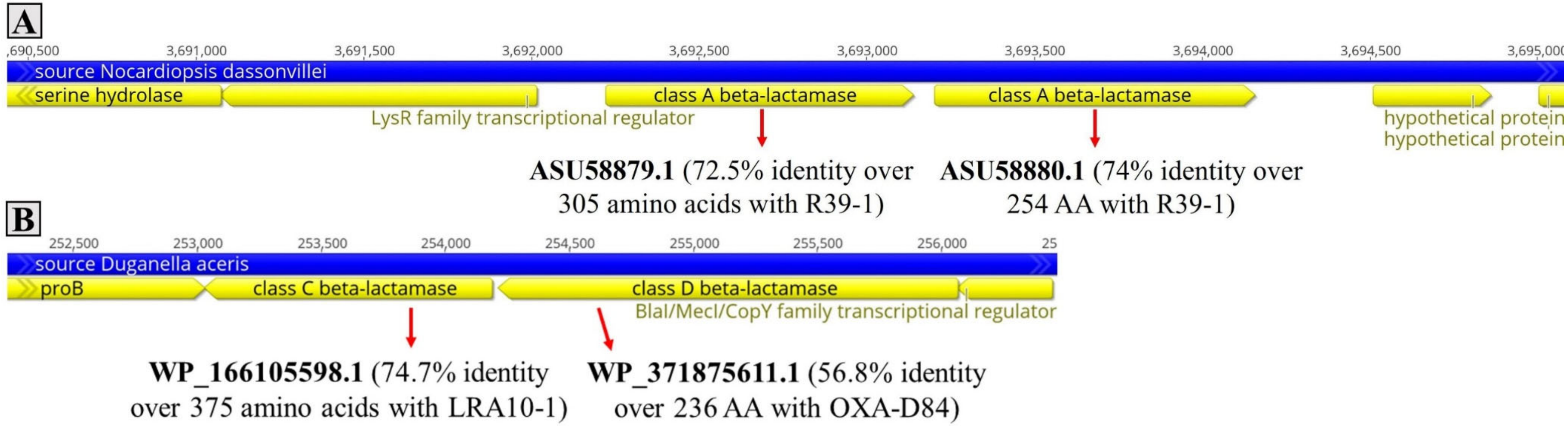

Detection of candidate beta-lactamases in *Nocardiopsis dassonvillei* (A) and *Duganella aceris* (B). Arrows indicate the direction of transcription. The length of the arrows is proportional to the size of the genes.
