# Supplementary material for: Are Putative Beta-Lactamases Posing a Potential Future Threat?
Source: Antibiotics (Basel). 2025 Nov 20;14(11):1174. doi: 10.3390/antibiotics14111174 (PMC12649608; doi:10.3390/antibiotics14111174)
Supplement: Supplementary file 1 [file antibiotics-14-01174-s001.zip › Supplementary Figure S2.pdf]

Supplementary Figure S2A. Circular, unrooted maximum-likelihood cladogram of class A beta-lactamases. The tree comprises 206 sequences (160 known beta-lactamase families from BLDB; 46 putative candidates, prefixed with “P-” and shown in bold). Branch labels display bootstrap proportions shown next to the node (reported as whole-number percentages). Because the cladogram transform is used, branch lengths are not proportional to evolutionary distance. Known-family tip labels are rendered in light gray for readability. Candidate identifiers and descriptions are listed in Table S1. Visualization in Geneious; inference details in Methods.

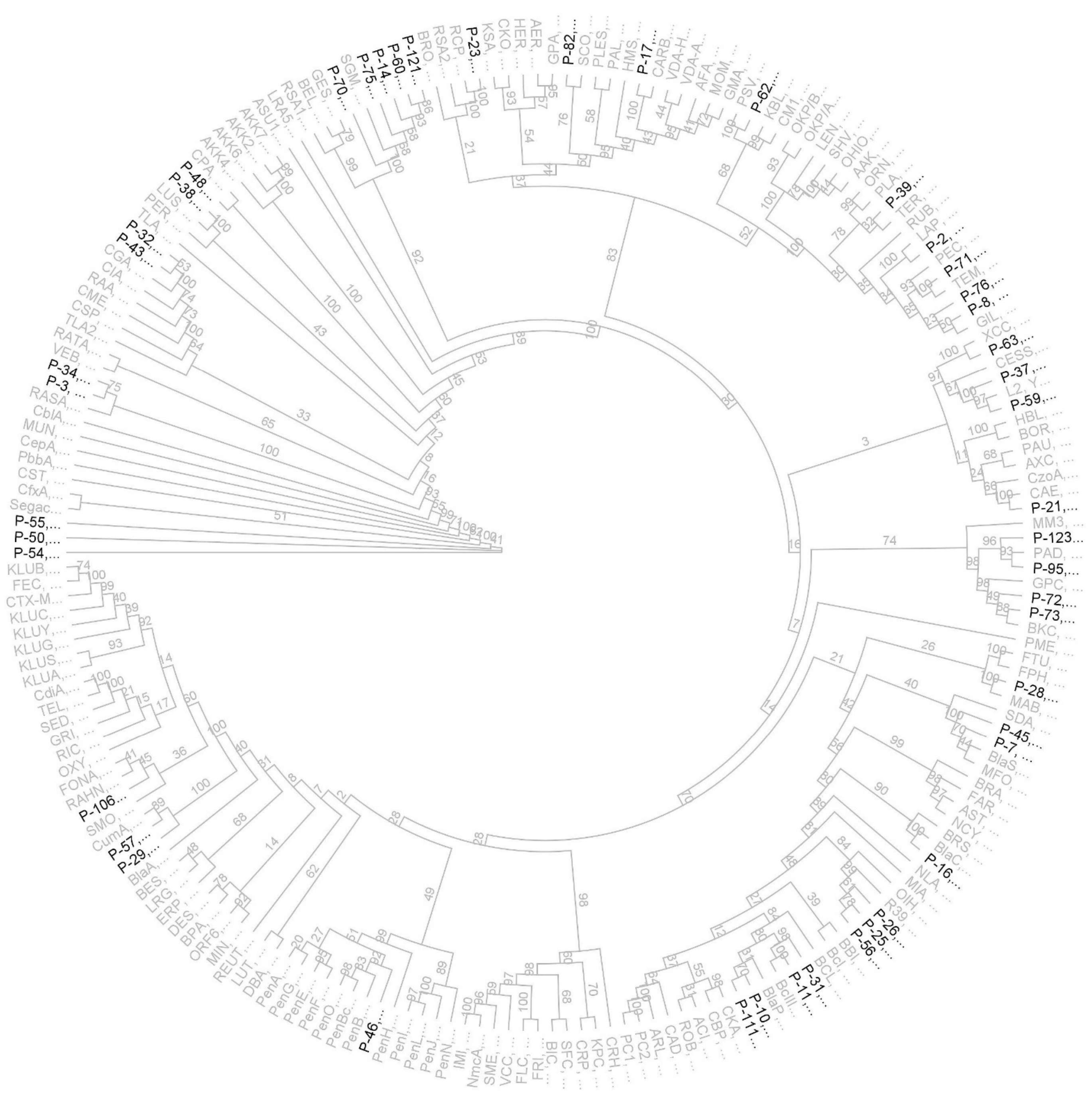

Supplementary Figure S2B. Circular, unrooted maximum-likelihood cladogram of class B beta-lactamases. The tree comprises 144 sequences (118 known beta-lactamase families from BLDB; 26 putative candidates, prefixed with "P-" and shown in bold). Branch labels display bootstrap proportions shown next to the node (reported as whole-number percentages). Because the cladogram transform is used, branch lengths are not proportional to evolutionary distance. Known-family tip labels are rendered in light gray for readability. Candidate identifiers and descriptions are listed in Table S1. Visualization in Geneious; inference details in Methods.

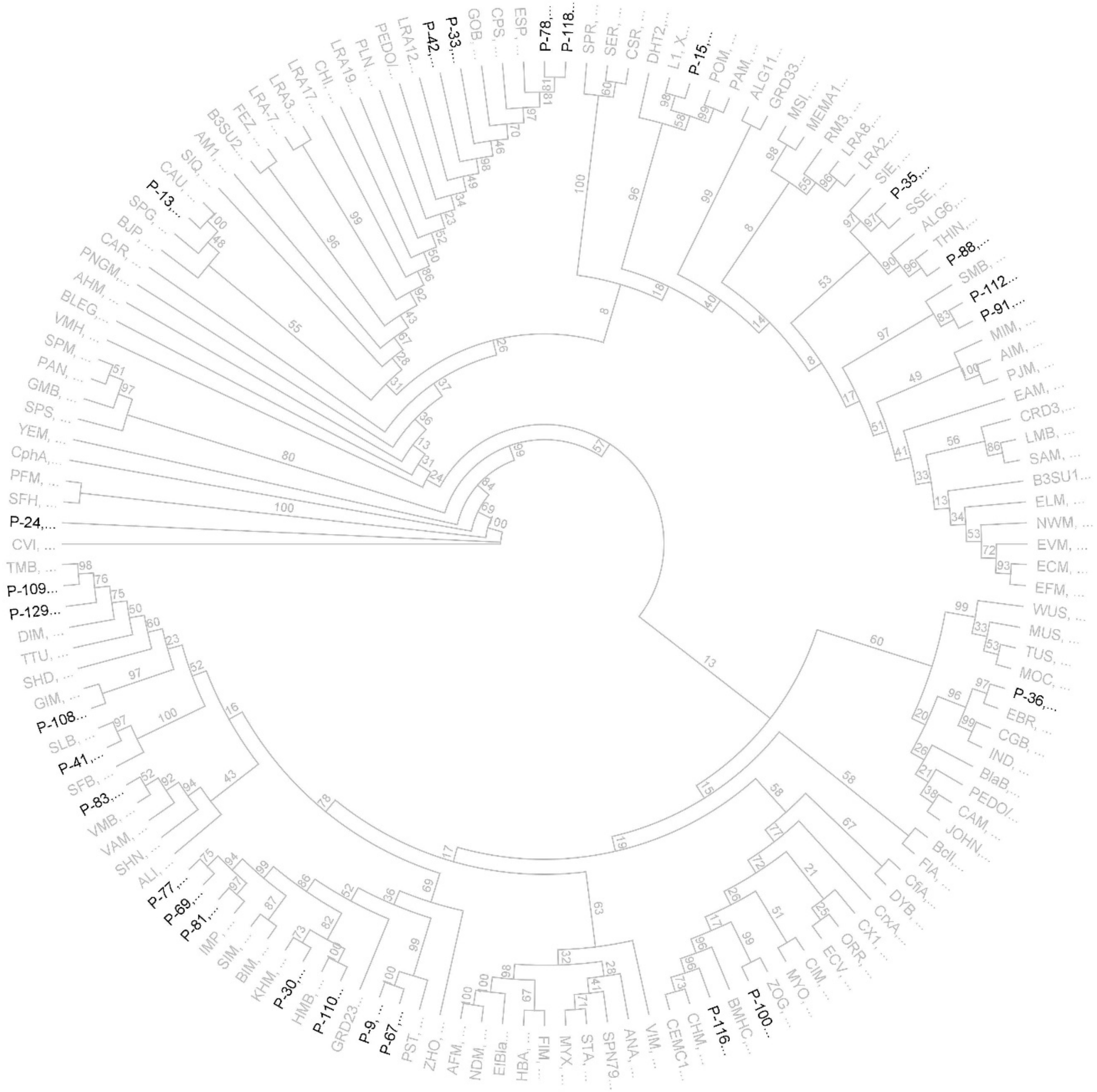

Supplementary Figure S2C. Circular, unrooted maximum-likelihood cladogram of class C beta-lactamases. The tree comprises 75 sequences (63 known beta-lactamase families from BLDB; 12 putative candidates, prefixed with "P-" and shown in bold). Branch labels display bootstrap proportions shown next to the node (reported as whole-number percentages). Because the cladogram transform is used, branch lengths are not proportional to evolutionary distance. Known-family tip labels are rendered in light gray for readability. Candidate identifiers and descriptions are listed in Table S1. Visualization in Geneious; inference details in Methods.

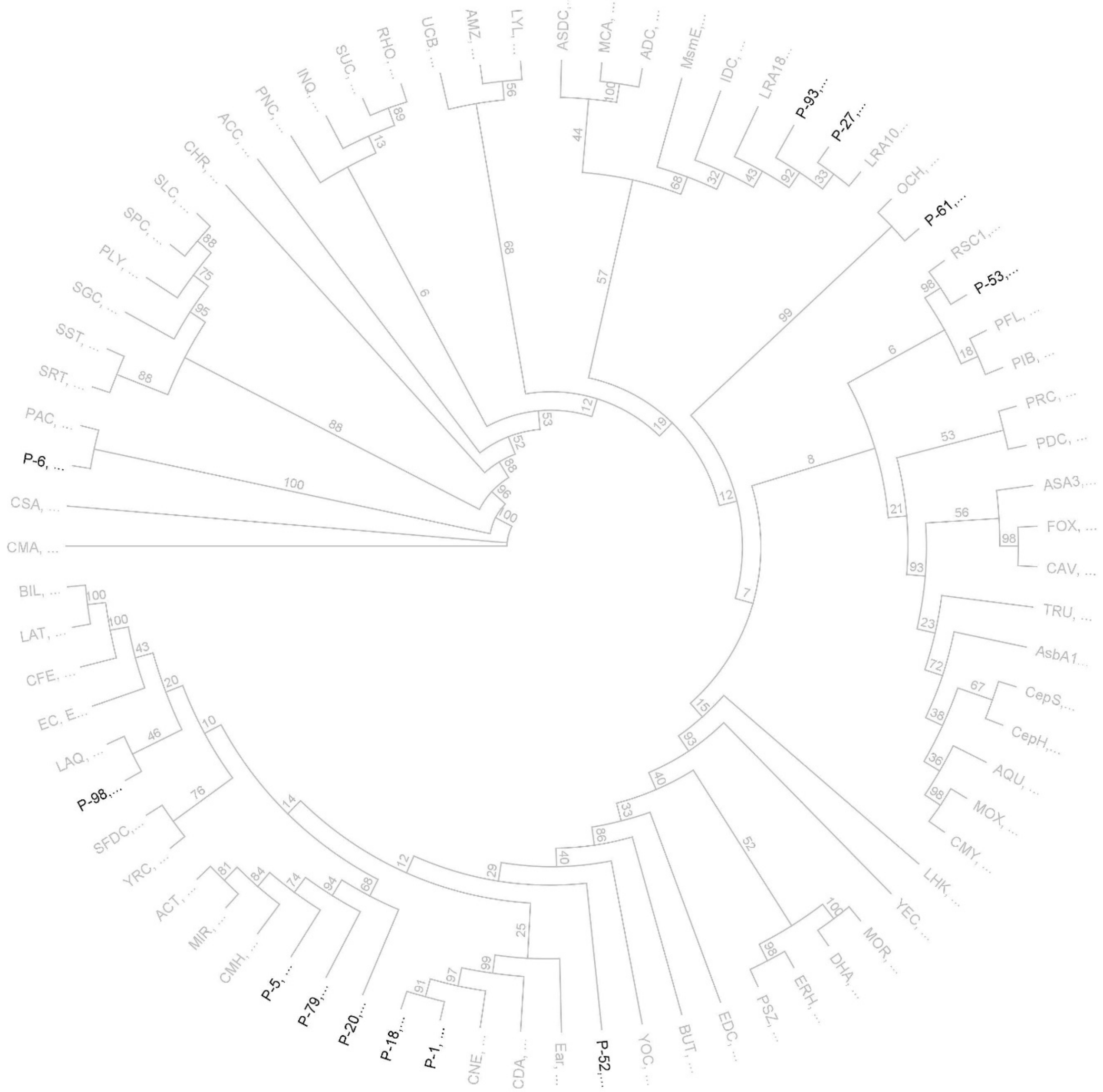

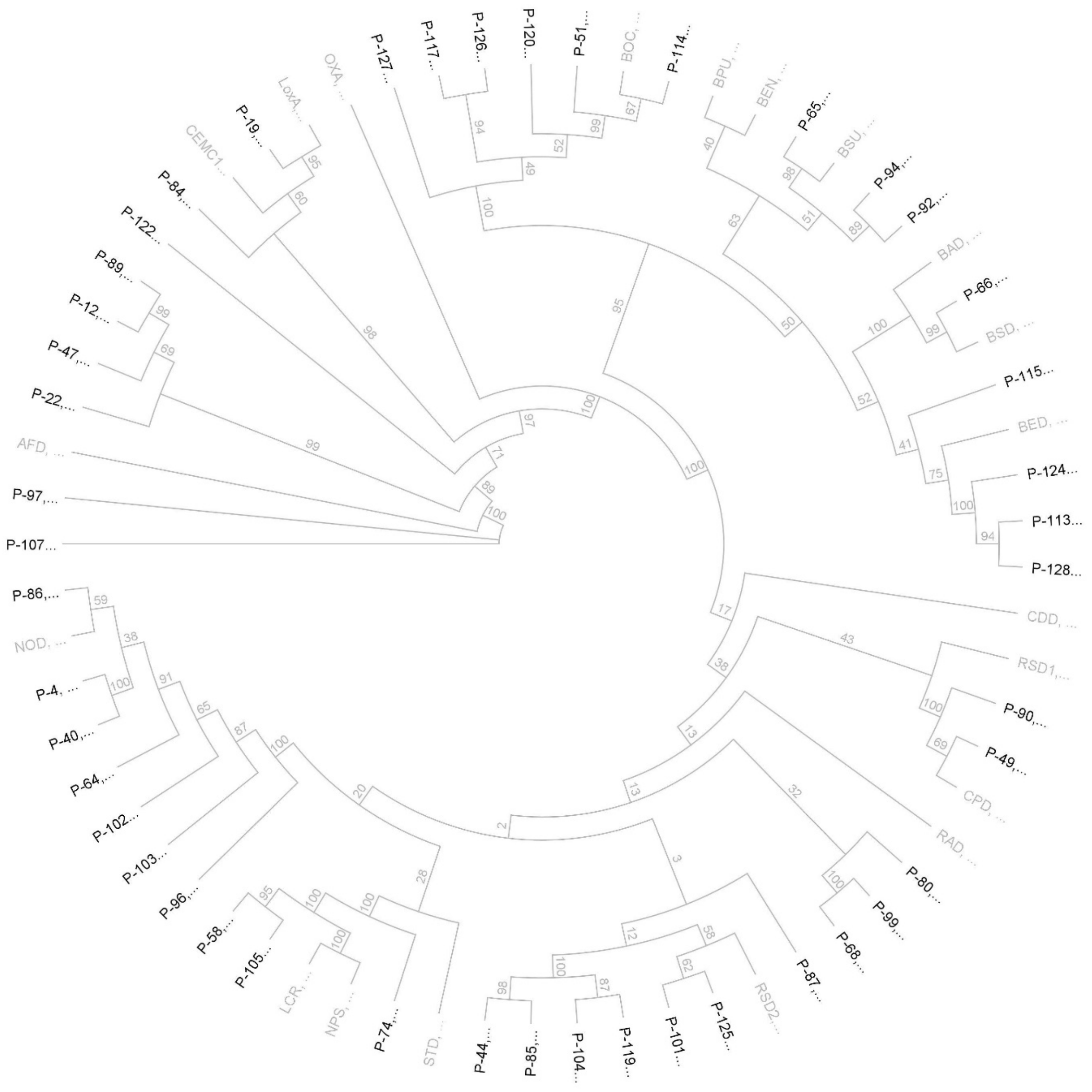

Supplementary Figure S2D. Circular, unrooted maximum-likelihood cladogram of class D beta-lactamases. The tree comprises 65 sequences (20 known beta-lactamase families from BLDB; 45 putative candidates, prefixed with "P-" and shown in bold). Branch labels display bootstrap proportions shown next to the node (reported as whole-number percentages). Because the cladogram transform is used, branch lengths are not proportional to evolutionary distance. Known-family tip labels are rendered in light gray for readability. Candidate identifiers and descriptions are listed in Table S1. Visualization in Geneious; inference details in Methods.
